# Geriatric Research, Education and Clinical Centers: Training Tomorrow’s Health Care Workforce

**DOI:** 10.1093/geroni/igaf122.736

**Published:** 2025-12-31

**Authors:** Marianne Shaughnessy

**Affiliations:** Veterans Health Administration, MIDDLE RIVER, Maryland, United States

## Abstract

Geriatric Research, Education and Clinical Centers (GRECC) are geriatric centers of excellence located throughout the U.S. One of the mandates of the GRECC program is to educate VA staff and trainees from their academic affiliates on evidence-based best practices in geriatric care. Over 45 years, GRECCs have educated thousands of students, residents and fellows in a range of health care professions, including, but not limited to: medicine, nursing, rehabilitation science, pharmacy, psychology, optometry, chaplaincy and social work. In fiscal year 2024, GRECCs collectively trained over 1600 medical students, residents and fellows, and over 400 Health Professions Trainees. GRECC educators introduce concepts of interdisciplinary and interprofessional team care informed by both the Geriatric 5Ms model, as an outgrowth of Age-Friendly Health Systems (AFHS), as well as the interprofessional education collaboration (IPEC) competencies. Finally, they have been a critical component of the spread of Age-Friendly Health Systems principles of care enterprise wide. Historically, each GRECC has developed and deployed its own educational curriculum. In 2022, the VA’s Office of Academic Affiliations made a formal request that GRECCs standardize their basic core curriculum in geriatrics. This presentation will introduce the GRECC interprofessional 5Ms core curriculum, discuss the educational resources in GRECCs and illuminate the creative and demonstrated achievements of GRECC educators. The vision for the curriculum was to meet the needs of a variety of health professions trainees organized around the Geriatric 5 Ms, (what matters, medication, mentation, mobility and multicomplexity) with considerations for former military not available in existing geriatric training materials.

